# Cholesterol affects the relationship between albumin and major adverse cardiac events in patients with coronary artery disease: a secondary analysis

**DOI:** 10.1038/s41598-022-16963-0

**Published:** 2022-07-25

**Authors:** Yu-Feng Yao, Zhen-Yu Chen, Tian-Yi Luo, Xiao-Yan Dou, Hai-Bo Chen

**Affiliations:** 1 Department of Ophthalmology, Shenzhen Second People’s Hospital, Shenzhen, 518035 Guangdong Province China; 2 Department of Cardiovascular Medicine, Shenzhen Second People’s Hospital, Shenzhen, 518035 Guangdong Province China; 3 Shantou University Medical College, No. 22 Xinling Road, 15724172356, Shantou, 515031 Guangdong Province China; 4 Shenzhen University, Shenzhen, 518037 Guangdong Province China

**Keywords:** Cardiovascular biology, Interventional cardiology

## Abstract

We aimed to examine whether the efficacy of the risk of poor prognosis in patients with coronary artery disease is jointly affected by total cholesterol and baseline serum albumin in a secondary analysis of previous study. We analyzed the data of 204 patients from October 2014 to October 2017 for newly diagnosed stable CAD. The outcome was major adverse cardiac events (MACE; defined as all cause mortality, non fatal myocardial infarction, and non fatal stroke). The median duration of follow-up was 783 days. Multivariable COX model was performed to revalidate the relationship between the sALB and MACE and interaction tests were conducted to find the effects of total cholesterol on their association. A total of 28 MACE occurred among the 204 participants. The risk of MACE varied by baseline serum albumin and total cholesterol. Specifically, lower serum albumin indicated higher risk of MACE (HR 3.52, 95% CI 1.30–9.54), and a test for interaction between baseline serum albumin and total cholesterol on MACE was significant (P = 0.0005). We suggested that baseline serum albumin and total cholesterol could interactively affect the risk of poor prognosis of patients with coronary artery diseases. Our findings need to be confirmed by further randomized trials.

## Introduction

Percutaneous coronary intervention (PCI), as an important strategy for cardiac revascularization in acute/chronic coronary artery disease (CAD), has been widely used in clinical practice. Compared with people without CAD, the CAD patients usually show higher long-term cardiovascular events, long-term revascularization events, and rehospitalization rates^1,2^. Some biomarkers such as high sensitivity cardiac troponin (hs-cTn), C-reactive protein (CRP), etc. are considered to be related to the long-term poor prognosis of CAD^3,4^. For patients with acute coronary syndrome (ACS) undergoing PCI, history of diabetes mellitus, uric acid level, blood urea nitrogen level, and activated partial thromboplastic time (APTT) were all considered risk factors of poor prognosis^5^. Compared with these biomarkers, serum albumin (sALB) has more and more been focused in recent years. In 2020, a study based on the CGPS (Copenhagen General Population Study) noted that lower sALB levels may be associated with an increased risk of long-term cardiovascular disease in healthy individuals^6^. In addition, some studies have suggested that low sALB levels may be associated with poor cardiovascular outcomes in patients with CAD^7–10^. Some previous studies have suggested that total cholesterol levels (TC) and low-density lipoprotein cholesterol levels (LDL-C), are risk factors for poor prognosis in CAD^12–14^. Moreover, some studies have shown that albumin is closely related to the metabolism and transport of cholesterol [23288948, 29685194, 35737216]. Therefore, we speculated that TC and serum albumin levels may jointly affect poor prognostic risk in patients with CAD. However, to date, this hypothesis has not been fully evaluated. To address this gap in knowledge, our study aimed to examine whether poor prognostic risk in patients with CAD is jointly affected by TC and serum albumin levels in baseline.

## Results

### Baseline characteristics of participants

The characteristics of the participants are shown in Table 1. Compared with the high sALB group (≥ 4 g/dL), the low sALB group (< 4 g/dL) had lower levels of BMI, Hb, T-chol, TG, LDL, eGFR, ALT, LVEF, and higher CRP level. Given that the low sALB group were older, the lower levels of BMI, sALB, lipoprotein, eGFR, LVEF and liver enzyme might be associated with malnutrition because of aging.

**Table 1:** Baseline characteristics of the study participants.

Variable	Serum albumin (g/dL)		P-value
	< 4 (n = 89)	≥ 4 (n = 115)	
Age (years)*	75.96 ± 10.87	69.98 ± 9.15	< 0.001
Male sex, n (%)^#^	59 (66.29%)	83 (72.17%)	0.365
BMI (kg/m^2^)^‡^	21.60 (4.49)	24.35 (3.78)	< 0.001
Systolic blood pressure (mmHg)^‡^	135.00 (25.00)	139.00 (21.50)	0.080
Diastolic blood pressure (mmHg)*	76.07 ± 15.21	78.48 ± 11.38	0.197
Hypertension, n (%)^#^	58 (65.17%)	93 (80.87%)	0.011
Dyslipidemia, n (%)^#^	32 (35.96%)	72 (62.61%)	< 0.001
Diabetes mellitus, n (%)^#^	33 (37.08%)	40 (34.78%)	0.734
Atrial fibrillation, n (%)^#^	14 (15.73%)	12 (10.43%)	0.261
Old cerebral infarction, n (%)^#^	19 (21.35%)	16 (13.91%)	0.162
PAD, n (%)^#^	27 (30.34%)	26 (22.61%)	0.212
Past smoker, n (%)^#^	34 (38.20%)	67 (58.26%)	0.004
LVEF (%)^‡^	65.00 (9.70)	66.95 (4.20)	0.014
Hb (g/dL)^‡^	12.40 (2.70)	14.60 (1.90)	< 0.001
TC (mg/dL)^*^	174.62 ± 34.60	197.24 ± 34.54	< 0.001
TG (mg/dL)^‡^	95.00 (65.00)	131.00 (100.50)	< 0.001
HDL-C (mg/dL)*	49.09 ± 14.03	52.60 ± 13.81	0.075
LDL-C (mg/dL)*	99.44 ± 27.76	117.56 ± 27.07	< 0.001
eGFR (mL/min/1.73 m^2^)^‡^	57.00 (35.00)	67.00 (19.50)	< 0.001
AST (U/L)^‡^	22.00 (12.00)	23.00 (9.00)	0.195
ALT (U/L)^‡^	16.00 (13.00)	20.00 (13.00)	< 0.001
HbA1C (%)^‡^	6.00 (1.20)	6.00 (1.00)	0.501
CRP (mg/dL)^‡^	0.25 (0.78)	0.07 (0.12)	< 0.001
Aspirin, n (%)^#^	87 (97.75%)	115 (100.00%)	0.189
Thienopiridines, n (%)^#^	87 (97.75%)	113 (98.26%)	0.795
Warfarin, n (%)^#^	2 (2.25%)	3 (2.61%)	0.868
DOAC, n (%)^#^	11 (12.36%)	10 (8.70%)	0.393
Ezetimibe, n (%)^#^	1 (1.12%)	2 (1.74%)	0.717
PPI, n (%)^#^	57 (64.04%)	77 (66.96%)	0.664
Statins, n (%)^#^	39 (43.82%)	72 (62.61%)	0.008
ACE-I, n (%)^#^	9 (10.11%)	10 (8.70%)	0.730
ARB, n (%)^#^	40 (44.94%)	48 (41.74%)	0.647
β-blocker, n (%)^#^	25 (28.09%)	30 (26.09%)	0.749
MRA, n (%)^#^	6 (6.74%)	5 (4.35%)	0.453
Multivessel disease, n (%)^#^	26 (29.21%)	27 (23.48%)	0.354
LMT lesions, n (%)^#^	8 (8.99%)	5 (4.35%)	0.178
Calcified lesions, n (%)^#^	20 (22.47%)	9 (7.83%)	0.003
Ostial lesions, n (%)^#^	15 (16.85%)	15 (13.04%)	0.446
Bifurcation lesions, n (%)^#^	45 (50.56%)	57 (49.57%)	0.888
CTO lesions, n (%)^#^	3 (3.37%)	9 (7.83%)	0.180
DES use, n (%)^#^	83 (93.26%)	110 (95.65%)	0.453
BMS use, n (%)^#^	6 (6.74%)	5 (4.35%)	0.453

*MACE* major adverse cardiac events (Defined as all-cause death, non-fatal myocardial infarction, non-fatal stroke), *BM* body mass index, *PAD* Peripheral artery disease, *Hb* hemoglobin, *TC* total cholesterol, *TG* Triglycerides, *HDL-C* high-density lipoprotein cholesterol, *LDL Chol* low density lipoprotein cholesterol, *eGFR* estimated glomerular filtration rate, *HbA1c* hemoglobin A1c, *CRP* C-reactive protein, *LVEF* left ventricular ejection fraction, *DOAC* direct oral anticoagulants, *PPI* proton pump inhibitor, *ACE-I* angiotensin converting enzyme inhibitor, *ARB* angiotensin-receptor blocker, *MRA* mineralocorticoid receptor antagonist, *LMT* left main trunk, *CTO* chronic total occlusion, *DES* drug-eluting stent, *BMS* bare metal stent.

*For continuous variables with normal distribution, values are presented as Mean ± SD.

^‡^For continuous variables with abnormal distribution, values are presented as Median (IQR).

^#^For categorical variable, values are presented as N (%).

### Relationship between the sALB and MACE

**Figure 1:**
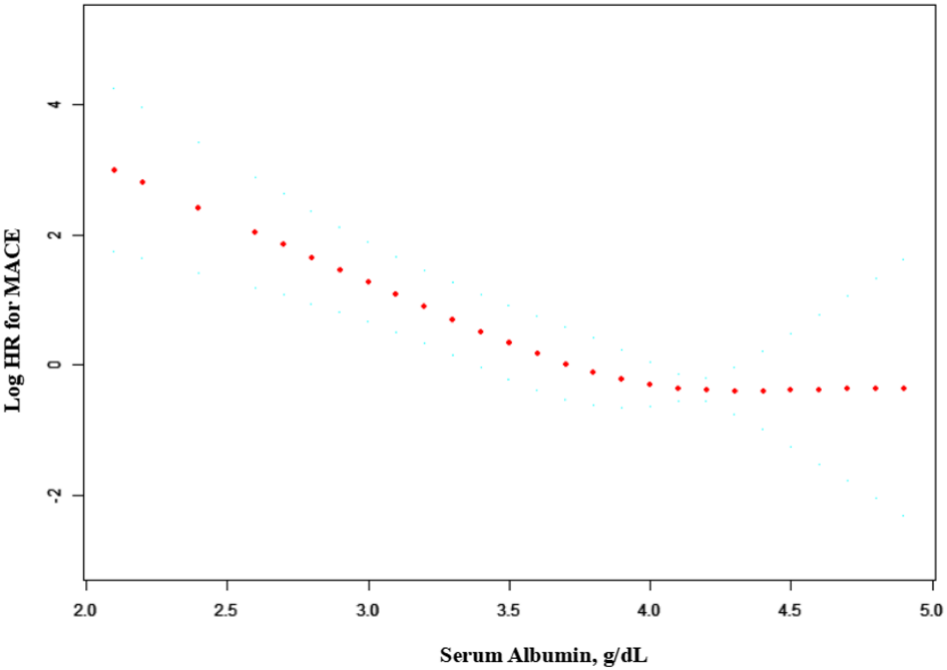
Smooth curves between sALB and MACE. The graph displays the adjusted association between sALB and the risk of MACE. The model adjusted for Age; MALE (sex); BMI; ALT; T-chol; eGFR.

Among the 203 participants included in the analyses, a total of 28 MACE occurred, including 22 in the low sALB group and 6 in the high sALB group. Cox model was used to evaluate the association between sALB and the risk of MACE. After fully adjusting for covariates, the HR of MACE was 0.22 (95% CI 0.11–0.44, P < 0.0001) (Table 2). Cox models were established by dividing the sALB of participants into the low sALB group (< 4 g/dL) and the high sALB group (≥ 4 g/dL) according to the median level. The results showed that, compared with the high sALB group, the HR of MACE in the low sALB group was 3.52 (95% CI 1.30–9.54, P = 0.0133) in the adjusted model. which did not change markedly comparing with the previous results^15^. Figure 1 showed a trend in the association between sALB and the risk of MACE (logHR), indicating that the risk of MACE decreased as the baseline sALB increased. When sALB level was more than 4.0 g/dL, it remained a protective factor and the risk of MACE remained unchanged.

**Table 2:** Relationship between sALB and MACE in adjusted model.

sALB, g/dL	n	Events, n (%)	HR (95%CI)	P value
As linear trend	203	28	0.22 (0.11, 0.44)	< 0.001
**As median cutoff point**				
Higher (≥ 4)	114	6	Reference	0.0133
Lower (< 4)	89	22	3.52 (1.30, 9.54)	

Adjusted for: Age; MALE (sex); BMI; ALT; T-chol; eGFR.

### Statistical interaction between sALB and TC on MACE

**Figure 2:**
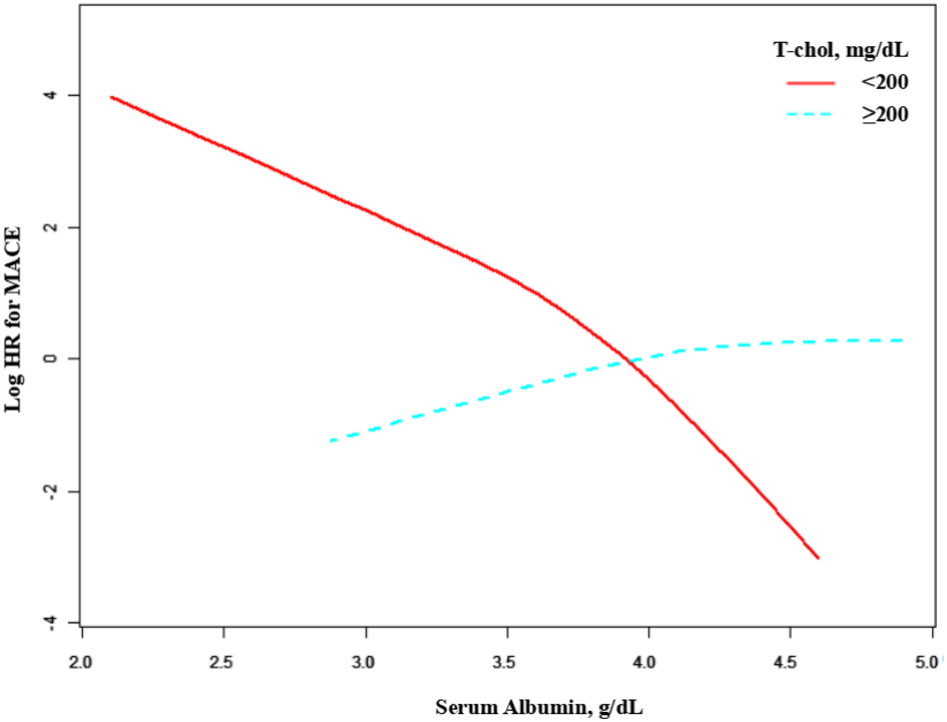
Smooth curves between sALB and MACE stratified by TC. The graph displays the adjusted association between sALB and the risk of MACE stratified by TC. The model adjusted for Age, MALE (sex), BMI, ALT, eGFR.

Figure 2 presents the association between sALB level and the risk of MACE (logHR), stratified by TC. The solid line, which represents those whose TC less than 200 mg/dL, shows a decline in MACE risk with increasing sALB level after adjusting for covariates. Contrarily, there is no such association in those whose TC more than 200 mg/dL. Because of sample size limitation, our primary outcomes were the tests for interaction between sALB and TC on MACE. The result was statistically significant in crude model (P = 0.0005), and compared with crude model, the effect of TC on the relationship between sALB and MACE did not change markedly after adjusting for age, sex, BMI, ALT, eGFR in the multivariable analysis (P = 0.0005) (Table 3).

**Table 3:** Effect modification of TC on the relationship between sALB and MACE.

	n	Events, %	HR (95% CI)	P for interaction
**Crude model**				
sALB^‡^ TC < 200 mg/dL	132	20 (15.15)	0.07 (0.03, 0.17)	0.0003
sALB^‡^ TC ≥ 200 mg/dL	72	8 (11.11)	1.15 (0.27, 4.99)	
Adjusted model*				
sALB^‡^ TC < 200 mg/dL	131	20 (15.27)	0.08 (0.03, 0.21)	0.0005
sALB^‡^ TC ≥ 200 mg/dL	72	8 (5.36)	1.28 (0.28, 5.77)	

*Adjusted for: Age, MALE (sex), BMI, eGFR, ALT. ^‡^Interaction test.

## Discussion

As some previous studies reported, baseline TC and sALB seem to be risk factors of poor prognosis in CAD patients. We conducted this study mainly to explore whether there is an interaction between them. We analyzed the interaction between TC and sALB and adjusted for covariates that may have influenced the results based on literature reports and clinical practice. Our analysis showed an interaction between TC and sALB levels after adjusting for covariates, which may suggested that baseline TC may influence the effect of sALB on the risk of poor prognosis in patients with coronary artery disease undergoing percutaneous coronary intervention. The relationship between sALB and cardiovascular risk has ever been reported by some previous studies. The study by Suzuki et al. suggested that the baseline sALB was a risk factor of long-term MACE in patients undergoing PCI^15^. Shih-Chieh Chien et al. pointed out that sALB is one of the reliable risk factors of CVD prognosis^16^. Oduncu et al. retrospectively analyzed 1706 patients with ST-segment elevation myocardial infarction (STEMI) who underwent PCI and suggested that lower sALB was independent risk factor of long-term mortality^17^. The potential mechanisms linking TC with sALB in cardiovascular prognostic risk have not been clearly elucidated. The molecular mechanism of albumin's effect on the prognosis of CVD may be multifactorial, which may include maintaining vascular endothelial integrity, regulating vasodilation, binding toxins, anticoagulation, and antioxidation, etc.^7^. Burl R Don et al. pointed out that the poor nutritional status reflected by low sALB may also be one of the risk factors of poor prognosis^18^. Studies have pointed out that albumin plays an important role in the transport of serum and cellular cholesterol and the maintenance of cholesterol homeostasis^11,16,19,20^. Albumin can shuttle free cholesterol among various receptors, which will increase the movement of free cholesterol under concentration gradients between the numerous cholesterol pools present in plasma and tissues, thereby promoting and maintaining cholesterol metabolism to restore steady-state levels^20^. The improving of cholesterol efflux may improve the prognosis of CAD^21^. What’s more, Deepak Kumar et al. found that the pseudoesterase activity of albumin might be an important determinant of cholesterol biosynthesis^22^. These study results may suggest that the influence of albumin level on the risk of poor prognosis may be closely related to cholesterol level. In conclusion, based on our analysis, we believe that TC level may influence the effect of sALB on the risk of poor prognosis in CAD patients treated by percutaneous coronary intervention. The potential limitations of the current analysis should also be considered in interpreting the study results. First, the data of our study came from a single-center sample, mainly elderly patients with CAD who underwent PCI. Therefore, for the extension of the research conclusions, further research is still needed. Second, the patients with chronic liver diseases, end stage renal diseases, malabsorption disease can effect serum albumin and cholesterol levels, but our data did not include these situation of the participants. Thus, we do not know whether TC affects the association of sALB with MACE in these patients, so our conclusions should be applied with caution in this group of patients. Finally, we have to emphasize that our results are merely hypothesis-generating; confirmation of our findings in an independent randomized trial is essential.

## Method

### Research design and population

This was a secondary scientific analysis of a retrospective, single-center cohort study where the dataset was collected by Sho et al. and is now available on Dryad (via:10.5061/dryad.fn6730j)^15^. As previously described, the cohort included hospitalized patients newly diagnosed with stable coronary artery disease and treated with PCI at Shinonoi general hospital from October 2014 to October 2017 in Shinonoi General Hospital. Patients with old myocardial infarction or malignancy were excluded. All participants were enrolled after the approval of Shinonoi General Hospital Ethics Committee and written informed consent. The research was consistent with the principles outlined in Helsinki Declaration. The outcome was major adverse cardiac events (MACE; defined as all cause mortality, non fatal myocardial infarction, and non fatal stroke). Events were validated by chart review and the median duration of follow-up was 783 days^15^. In our analysis, we included all the cases to revalidate the relationship between the sALB and MACE by adjusting more covariates than the previous research^15^, and more importantly, we tried to find the effects of TC on their association by interaction tests.

### Statistical analysis

The baseline characteristics of the participants were analyzed and divided into two groups according to the median baseline sALB level. The continuous variables with normal distribution were presented as Mean ± SD (comparison between groups by t test), the continuous variables with abnormal distribution were presented as Median (IQR) (comparison between groups by Kruskal Wallis rank sum test) and the categorical variables were presented as N (%) (comparison between groups by χ^2^ test).

The primary outcome was to determine the effect of TC on the association between MACE and baseline sALB level. Therefore, the results of unadjusted and adjusted covariates model analysis were presented based on STROBE recommendations. When the covariate was included or excluded from the model, the hazard ratio of the match was changed by at least 10%, and this covariate needed to be adjusted^23^. Besides, if it was related to serum albumin and MACE in clinical practice, covariate wound be included with other congener covariates excluded. Moreover, covariates adjusted in previous congener studies were also included^24^. And finally we adjusted, if not stratified, for age, sex, BMI, TC, eGFR, ALT in the multivariable model.

In the multivariable regression analysis, baseline sALB level was first analyzed as a continuous variable and then divided into two groups according to the median level. Interaction tests were conducted according to TC (< 200 mg/dL and ≥ 200 mg/dL), and the models were divided into crude model and adjusted model according to the adjusted covariates.

P values less than 0.05 (two-tailed) were considered statistically significant. EmpowerStats ver.3.0 (http://www.empowerstats.net/analysis/, X&Y Solutions,Inc., Boston, MA) and the R software ver.3.3.1 (http://www.R-project.org/, The R Foundation), were used for all statistical analyses.

## Data Availability

The dataset was collected by Sho et al. and is now available on Dryad (via: 10.5061/dryad.fn6730j). The datasets generated or analysed during the current study are available from the corresponding author on reasonable request.
